# Bub3 Is a Spindle Assembly Checkpoint Protein Regulating Chromosome Segregation during Mouse Oocyte Meiosis

**DOI:** 10.1371/journal.pone.0007701

**Published:** 2009-11-02

**Authors:** Mo Li, Sen Li, Ju Yuan, Zhen-Bo Wang, Shao-Chen Sun, Heide Schatten, Qing-Yuan Sun

**Affiliations:** 1 State Key Laboratory of Reproductive Biology, Institute of Zoology, Chinese Academy of Sciences, Beijing, China; 2 Graduate School, Chinese Academy of Sciences, Beijing, China; 3 Department of Veterinary Pathobiology, University of Missouri-Columbia, Columbia, Missouri, United States of America; University of Hong Kong, Hong Kong

## Abstract

In mitosis, the spindle assembly checkpoint (SAC) prevents anaphase onset until all chromosomes have been attached to the spindle microtubules and aligned correctly at the equatorial metaphase plate. The major checkpoint proteins in mitosis consist of mitotic arrest-deficient (Mad)1–3, budding uninhibited by benzimidazole (Bub)1, Bub3, and monopolar spindle 1(Mps1). During meiosis, for the formation of a haploid gamete, two consecutive rounds of chromosome segregation occur with only one round of DNA replication. To pull homologous chromosomes to opposite spindle poles during meiosis I, both sister kinetochores of a homologue must face toward the same pole which is very different from mitosis and meiosis II. As a core member of checkpoint proteins, the individual role of Bub3 in mammalian oocyte meiosis is unclear. In this study, using overexpression and RNA interference (RNAi) approaches, we analyzed the role of Bub3 in mouse oocyte meiosis. Our data showed that overexpressed Bub3 inhibited meiotic metaphase-anaphase transition by preventing homologous chromosome and sister chromatid segregations in meiosis I and II, respectively. Misaligned chromosomes, abnormal polar body and double polar bodies were observed in Bub3 knock-down oocytes, causing aneuploidy. Furthermore, through cold treatment combined with Bub3 overexpression, we found that overexpressed Bub3 affected the attachments of microtubules and kinetochores during metaphase-anaphase transition. We propose that as a member of SAC, Bub3 is required for regulation of both meiosis I and II, and is potentially involved in kinetochore-microtubule attachment in mammalian oocytes.

## Introduction

To assure accurate chromosome segregation during mitosis, eukaryotic cells employ the spindle assembly checkpoint (SAC) mechanism to prevent premature progression to anaphase until all chromosomes are successfully attached to the bipolar spindle with the proper tension. Prior to metaphase-anaphase transition, unattached kinetochores contribute to the formation of the mitotic checkpoint complex (MCC), containing mitotic arrest-deficient (Mad)2, budding uninhibited by benzimidazoles related 1 (BubR1/Mad3) and budding uninhibited by benzimidazole (Bub)3, as well as Cdc20 itself [1], which has been defined in recent years as a possible SAC effector, and which inhibits the ability of Cdc20 to activate the anaphase-promoting complex/cyclosome (APC/C), stabilizes securin and cyclin B, and thus delays the metaphase-anaphase transition until all chromosomes have established the correct attachment to the spindle [2], [3]. Once the SAC is inactivated, APC/C-Cdc20 ubiquitinates securin and cyclin B, leading to the activation of separase which removes the cohesion complex so that the cells can enter anaphase [4], [5], [6]. In meiosis, two successive divisions occur with only one round of DNA replication, which thereby produces haploid gametes. Errors in chromosome segregation in mitotic cells lead to aneuploidy and tumor progression [7], [8], [9], while errors in chromosome segregation in meiotic germ cells lead to development failure or abortion [10], [11], [12].

The major checkpoint proteins in mitosis consist of Mad1–3, Bub1, Bub3, and monopolar spindle 1 (Mps1) [13], [14], [15]. Besides, motor proteins (CENP-E, MCAK, and dynein) and kinases (MAPK, auroraB) are also recruited to unattached kinetochores and are important factors for the spindle checkpoint [2], [10], [16]. Subcellular localization studies have placed most checkpoint proteins at the kinetochores and some of them (Mad1, Mad2, Bub1, Bub3 and BubR1) are present only at unattached rather than at fully microtubule-attached kinetochores [17], [18], [19], [20]. How the SAC detects the errors and controls the cell cycle is complex and still puzzling.

It is worthwhile noting that the existence of the SAC is controversial [10], [21], [22] and the related molecular mechanisms are unclear in oocyte meiosis. In mitosis and meiosis II, sister kinetochores attach to microtubules in an amphitelic manner and sister chromatids are segregated to opposite poles during the metaphase-anaphase transition due to destruction of the residual cohesion holding sister centromeres together [10], [23]. However, in meiosis I, sister kinetochores attach to microtubules with the same polarity (syntelic attachment), which is called mono-orientation [21], [24], [25], and homologous chromosomes segregate with monopolar kinetochore orientation, which in mitosis is regarded as a faulty attachment [23], [26].

As a core checkpoint component in the mitotic cell cycle, the accurate roles of Bub3 in meiosis are little known. The major contribution of Bub3 as component of the MCC in mitosis [1] appears to be targeting of Bub1 and BubR1/Mad3 to kinetochores [27], [28], [29]. A recent study showed that Bub3 is required for the establishment of kinetochore-microtubule attachment in HeLa cells [30]. In the present study we produced Myc_6_-Bub3 mRNA to overexpress Bub3 in mouse oocytes and we also employed RNAi to suppress Bub3 function to address the role of Bub3 during meiosis. Cold treatment combined with Bub3 overexpression was used to study the interaction between kinetochore and spindle microtubules. The results indicate that overexpressed Bub3 inhibits metaphase-anaphase transition by preventing homologous chromosomes and sister chromatids segregations during meiosis I and II, respectively, and that loss of Bub3 function causes misaligned chromosomes and aneuploidy. Furthermore, Bub3 may be important for the attachment of microtubules to kinetochores. We propose that, as a checkpoint protein, Bub3 is required for mammalian oocytes to monitor the meiotic cell cycle and to ensure high fidelity of chromosome segregation.

## Results

### Expression and subcellular localization of Bub3 during meiosis

**Figure 1:**
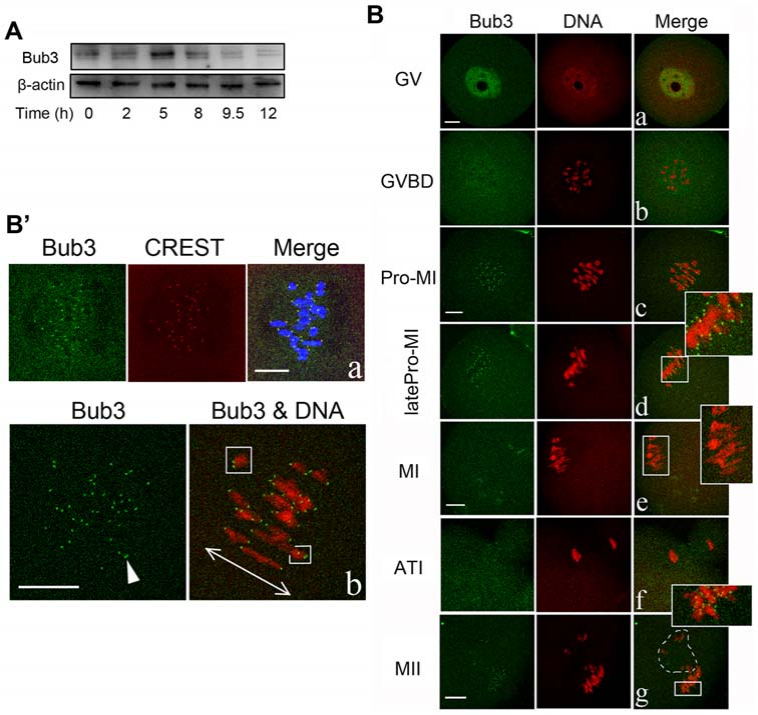
Expression and subcellular localization of Bub3 during oocyte meiosis. (A) Samples were collected after oocytes had been cultured for 0, 2, 5, 8, 9.5, 12 h, corresponding to GV, GVBD, Pro-MI, MI, AI, and MII stages, respectively. Proteins from 300 oocytes were loaded for each sample. The highest level of Bub3 expression was found at Pro-MI. (B) Oocytes at various stages were stained with anti-Bub3 antibody (green) and propidium iodide (PI, red). Clear signal of Bub3 was observed at kinetochores in Pro-MI, latePro-MI and MII stages. Dotted line area indicates PBI. Magnifications of the boxed regions are shown. b and c share the same bar; d and e share the same bar; f and g share the same bar. Bar  = 10 µm. (B′) a, co-localization of Bub3 and CREST at kinetochores. Green, Bub3; red, CREST; Blue, DNA. b, 40 dots of Bub3 signal were shown in oocytes. Arrowhead indicates 2 dots which are faintly visible. Boxed chromosomes are the homologues without proper attachment to MTs. Bi-directed arrow indicates the tension direction. Bar  = 10 µm.

To investigate the role of Bub3 during meiosis, we first examined the expression level and subcellular localization of this protein. Samples of mouse oocytes at different stages of meiosis were collected after oocytes had been cultured for 0, 2, 5, 8, 9.5, and 12 h, corresponding to germinal vesicle (GV), germinal vesicle breakdown (GVBD), prometaphase I (Pro-MI), Metaphase I (MI), anaphase/telophase I (ATI), and metaphase II (MII) stages, respectively. As shown in Fig. 1A, Bub3 was expressed throughout meiosis. The expression level was moderate at GV and GVBD stages, and reached the highest level at Pro-MI. At MI, Bub3 was reduced to a similar level as during the GVBD stage and was then further reduced during ATI and MII stages. For the subcellular localization of Bub3, oocytes were processed for immunofluorescent staining at different stages of meiosis. In GV stages, Bub3 was mainly localized in the germinal vesicle (nucleus) (Fig. 1Ba); weak signal was detected around the chromosomes at GVBD (Fig. 1Bb); when oocytes progressed to Pro-MI, clear staining was observed at the kinetochores (Fig. 1Bc). After Pro-MI, we defined a short stage shortly before MI as latePro-MI to study details of Bub3 localization because the Pro-MI-MI transition is a crucial time point for tracing the behavior of checkpoint proteins. At latePro-MI, the signal of Bub3 was still obvious at the kinetochores even though most of the chromosomes had moved to the equatorial plane (Fig. 1Bd), suggesting that even one or two unattached chromosome(s) could keep Bub3's localization at kinetochores. Once all chromosomes were aligned at the equatorial plane without any mistake, i.e. the cells had progressed to MI, Bub3 could no longer be detected at the kinetochores (Fig. 1Be). By this time the oocytes were ready to enter the AI stage. At the AI/TI stage, the signal for Bub3 was undetectable at kinetochores (Fig. 1Bf). At the MII stage, Bub3 returned to the kinetochores of chromosomes in both oocytes and the polar bodies (Fig. 1Bg). Moreover, co-localization of Bub3 and CREST was performed to further confirm the kinetochore localization of Bub3 (Fig. 1B′a). Notably, as shown in Fig. 1B′b, Bub3 stayed at all 40 kinetochores even though most but not all homologues were attached with tension before the MI stage. These data imply that Bub3 may contribute to the cell cycle surveillance as a checkpoint protein in mammalian meiosis.

### Overexpressed Bub3 inhibits metaphase-anaphase transition by preventing homologous chromosome segregation during meiosis I

**Figure 2:**
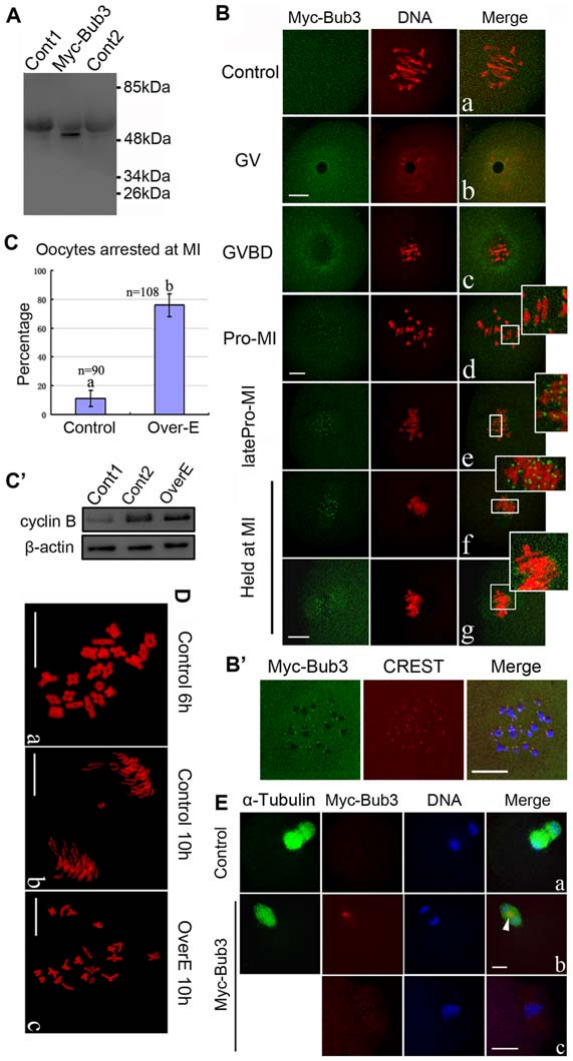
Overexpression of Bub3 in meiosis I inhibits MI-AI transition. (A) Samples from control and overexpression groups were collected to test the expression of Myc_6_-Bub3 mRNA. Cont1, 200 oocytes without injection; Myc-Bub3, 150 oocytes injected with 2.5 mg/ml Myc_6_-Bub3 mRNA solution; Cont2, 150 oocytes injected with the same amount of Myc_6_ mRNA. (B) Oocytes at various stages were stained with anti-Myc-FITC antibody (green) to detect the overexpressed-Bub3 and with PI (red) to visualize DNA. Control and overexpression groups were injected with the same amount of Myc_6_ mRNA and Myc_6_-Bub3 mRNA (the same below). Clear signal of overexpressed Bub3 was observed at kinetochores at Pro-MI, latePro-MI and MI stages. Overexpressed Bub3 arrested oocytes at MI stage by preventing homologues segregation. Magnifications of the boxed regions are shown. a and b share the same bar; c and d share the same bar; e–g share the same bar. (B′) co-localization of overexpressed Bub3 and CREST at kinetochores. Green, overexpressed Bub3; red, CREST; blue, DNA. (C) Effect of overexpressed Bub3 on MI-AI transition. Percentage of oocytes arrested at MI is shown by mean±SEM. Different superscripts indicate statistical difference (*P*<0.05). (C′) Expression levels of cyclin B in ATI stage oocytes (Cont1, cultured for 10 hours), MI stage oocytes (Cont2, cultured for 8 hours), and overexpressed oocytes (OverE, cultured for 10 hours). Each sample contained 300 oocytes. (D) Chromosome spreading was performed in oocytes of control and overexpression groups. Bivalents are shown in a and c; univalents are shown in b. (E) A few oocytes in the overexpression group overcame the inhibition and showed positive signal of overexpressed Bub3. Arrowhead indicates the overexpressed Bub3 in AI. Green, α-tubulin; red, overexpressed Bub3; blue, DNA. a and b share the same bar. Bar  = 10 µm.

To characterize the role of Bub3 in SAC during meiosis I, Bub3 overexpression experiments were performed. First, to confirm the overexpression, oocytes injected with the same amount of control Myc_6_ mRNA (Cont2), Myc_6_-Bub3 mRNA or without injection (Cont1) were collected for Western blot with the anti-myc antibody. As presented in Fig. 2A, in control groups, no specific blot was detected while in the overexpression group, a clear Myc_6_-Bub3 blot could be observed (40 (Bub3) kDa + approximate 10 (Myc) kDa  =  approximate 50 kDa), indicating the expression of Myc_6_-Bub3 mRNA.

Oocytes at the GV stage were injected with Myc_6_-Bub3 mRNA and then anti-myc-FITC antibody was used for immunofluorescent detection. The same amount of Myc_6_ mRNA was injected as control, but no specific signal was detected (Fig. 2Ba). As presented in Fig. 2Bb-g, Myc_6_-Bub3 mRNA was successfully overexpressed within the oocytes: in GV and GVBD stages, diffused signal of overexpressed Bub3 was detected in the GV and around the GVBD area, respectively. In Pro-MI and latePro-MI stages, overexpressed Bub3 was clearly localized at the kinetochores of chromosomes, which was the same as the endogenous Bub3 in Fig. 1Bc, d. During MI, however, overexpressed Bub3 was still present at the kinetochores and inhibited the transition from MI to AI by preventing the homologous chromosome segregation even though all chromosomes had been aligned at the equatorial plane and were ready for the AI transition (Fig. 2Bf); and the oocytes could not overcome the inhibition even when cultured for 10 hours (Fig. 2Bg), which was significantly different from that in Fig. 1Be. On the other hand, co-localization of overexpressed Bub3 and CREST was performed to further confirm their localization at kinetochores (Fig. 2B′). In the overexpressed group, most oocytes were not able to overcome the inhibition even if they were cultured for 10 hours, indicating that overexpressed Bub3 successfully inhibited the MI-AI transition during meiosis I (Fig. 2C). To confirm the oocyte arrested by overexpressed Bub3 at the MI stage, we also tested the level of cyclin B. As shown in Fig. 2C′, cyclin B's level in overexpressed oocytes was nearly the same as that in MI stage oocytes (Cont2) while it was much higher than that in AI stage oocytes (Contl). To examine the details of homologous chromosomes during the delayed MI-AI transition, we employed chromosome spreading. In meiosis, the number of bivalents in control oocytes cultured for 5–6 hours (Pro-MI) was 20 (Fig. 2Da). When oocytes entered AI, all bivalents separated and became univalents (Fig. 2Db). Notably, in the overexpression group, all oocytes (n = 12), whose MI-AI transition was successfully inhibited, displayed 20 bivalents, without any univalent (Fig. 2Dc). These results imply that overexpressed Bub3 inhibits the MI-AI transition and prevents homologous chromosomes from segregation in an “all or none” manner.

Among the overexpression oocytes cultured for 10 hours, a few (about 10% of the total oocytes) were able to enter AI even though the signal of overexpressed Bub3 was detected. But different from that in Fig. 2Bd-g, overexpressed Bub3 in this case was localized between the separated homologous chromosomes rather than at the kinetochores (Fig. 2Eb). In another overepression group, when cultured for 12 hours, no more than 13% oocytes (9/69) extruded the first polar body (PBI). Among these PBI extruded (PBE) oocytes, we found that some of them (5/9) exhibited an interesting segregation pattern of chromosomes. As shown in Fig. 2Ec, all of the homologous chromosomes were held together and entered the PBI. To confirm, we scanned the oocytes thoroughly in numerous planes and did not find chromosomes in any of these oocytes (see Fig. S1 in supplementary material).

### Overexpressed Bub3 inhibits metaphase-anaphase transition by preventing chromatids segregation during meiosis II

**Figure 3:**
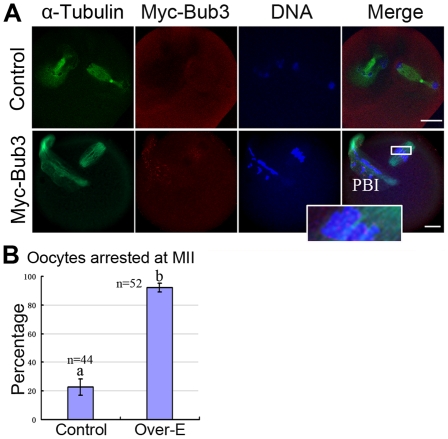
Overexpressed Bub3 inhibits the MII-AII transition by preventing sister chromatid segregation. MII oocytes were injected with Myc_6_ (control) or Myc_6_-Bub3 mRNA and cultured to the AII stage. (A) green, α-tubulin; red, overexpressed Bub3; blue, DNA. Magnifications of the boxed regions are shown. Bar  = 10 µm. (B) Percentages of oocytes arrested at MII in control and overexpression groups.

Since overexpressed Bub3 can inhibit the MI-AI transition during meiosis I, we wanted to know whether it also functions in meiosis II. To test this, MII oocytes were injected with Myc_6_-Bub3 and Myc (control) mRNA and cultured for 2 hours to allow the expression; the oocytes were then artificially activated. The results showed that overexpressed Bub3 was localized at kinetochores and the MII-AII transition was inhibited while in the control group, most of the oocytes entered the AII stage (Fig. 3A). Fig. 3B represents the different rates of oocytes arrested at the MII stage in control and overexpression groups. These data show that Bub3 participates in the MII-AII transition in meiosis II.

### Overexpressed Bub3 during MI-AI disrupts the attachment of microtubules to kinetochores

**Figure 4:**
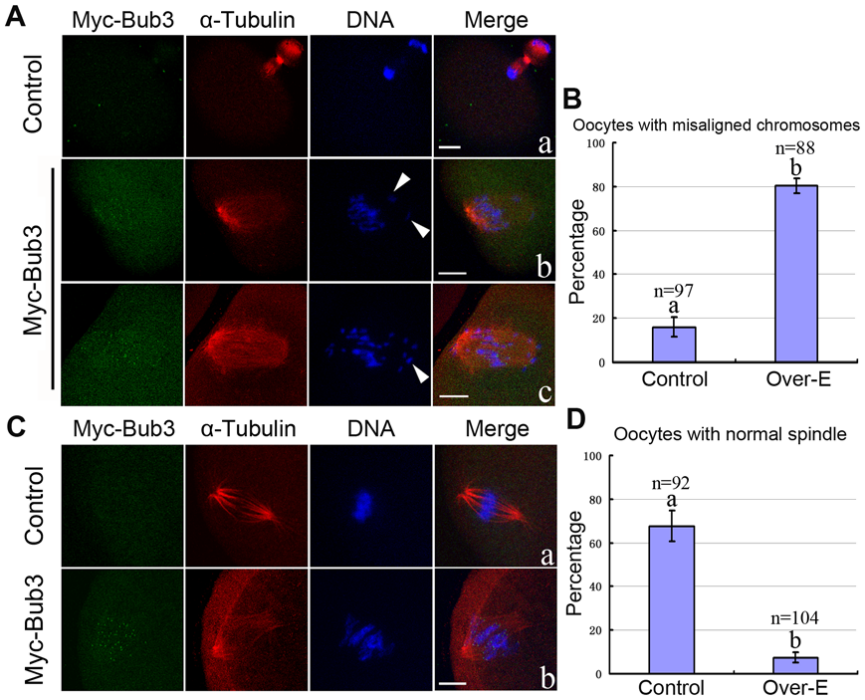
Overexpressed Bub3 interferes with the attachments between kinetochores and microtubules. (A) Myc_6_-Bub3 mRNA was expressed during MI-AI transition. Misaligned chromosomes were obvious in the overexpression group (b and c). Arrowhead indicates the severely misaligned homologues. Red, α-tubulin; green, overexpressed-Bub3; blue, DNA. (B) Percentages of oocytes with misaligned chromosomes in control and overexpression groups. (C) Oocytes of control and overexpression groups were cultured to the MI-AI transition or AI followed by cold treatment for 25 minutes at 4°C. Clustered and clear K-MTs were observed in control (a) but not in overexpression group, whereas abnormal spindle and misaligned chromosomes were observed in overexpression oocytes (b). Bar  = 10 µm. (D) Percentages of oocytes with normal spindles in control and overexpression groups.

Since the expression level of endogenous Bub3 was significantly reduced (Fig. 1A) at the AI stage and correspondingly, the immunocytochemical signal could not be detected (Fig. 1Bf), we wondered about the roles of overexpressed Bub3 at this stage. However, because overexpressed oocytes could not reach the AI stage (Fig. 2Bf-g and 2C), we therefore designed a new strategy to achieve Bub3 overexpression in AI: oocytes were first cultured for 7 hours, when most of them nearly but not completely had progressed to the MI stage. Then these oocytes were injected with the same amount of Myc_6_-Bub3 and control Myc_6_ mRNA (control) and cultured for 2 more hours. In our system, a 2-hours culture was sufficient to allow mRNA expression in most of the oocytes. So at 9 (7+2) hours, when most of the oocytes were undergoing or just completed the MI-AI transition with the overexpressed Bub3, we observed the oocytes with immunofluorescent microscopy. In the control group, oocytes entered the anaphase-telophase stage (ATI) (Fig. 4Aa). Notably, in the overexpression group, most of the oocytes were not able to enter the ATI stage. Among these oocytes, 32% were arrested at the MI-AI transition stage (Fig. 4Ab) and 48% appeared to be close to the AI stage (Fig. 4Ac). Both kinds of the oocytes contained misaligned homologous chromosomes in the spindle (Fig. 4Ab, Fig. 4Ac, and Fig. 4B). These results indicate that during the MI-AI transition, overexpressed Bub3 may affect kinetochore-microtubule attachment, leading to the incorrect chromosome segregation.

To further test our speculation, we performed cold-treatment of oocytes. Kinetochore microtubules (K-MTs) display different stability to cooling compared to non-kinetochore microtubules, i.e. free, polar, and astral spindle microtubules (MTs) disassemble while K-MTs cluster together and persist as bundles of cold-stable MTs [30], [31]. Oocytes cultured for 7 hours were injected with Myc_6_-Bub3 or control Myc_6_ mRNA followed by 2 more hours of culture when most of the oocytes expressed the mRNA and were undergoing or just completed the MI-AI transition. Then the oocytes were cultured at 4°C for 25 minutes, and subsequently used for immunofluorescent staining. As expected, in the control group, K-MTs but not free MTs were cold-stable and clustered together (Fig. 4Ca). Whereas in the overexpression group, feeble and amorphic MTs (spindle) but few K-MTs were observed (Fig. 4Cb). Statistical analysis is shown in Fig. 4D for these oocytes. The results imply that overexpressed Bub3 appears to break the attachment between kinetochores and microtubules during the MI-AI transition.

### Deletion of Bub3 by RNAi abrogates the metaphase arrest induced by nocodazole

**Figure 5:**
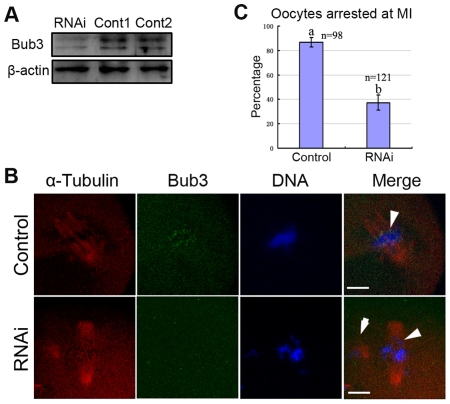
Bub3-RNAi abrogates oocyte meiotic arrest induced by nocodozale. (A) Samples from control and RNAi groups were collected to test the efficiency of Bub3-RNAi. RNAi, 300 oocytes injected with 25 µM siRNAs; Cont 1, 300 oocytes without injection; Cont 2, 300 oocytes injected with 25 µM control siRNAs. (B) and (C) control (injected with control siRNAs), nocodozale induced oocyte arrest at MI stage; RNAi, Bub3-RNAi oocytes broke through the MI arrest induced by nocodozale and reached the MII stage. Arrowheads in control and RNAi groups indicate Bub3 signal and misaligned chromosomes, respectively. Arrow in RNAi group indicates the PBI. Red, α-tubulin; green, Bub3; blue, DNA. Bar  = 10 µm.

We further investigated the effects of Bub3 down-regulation on meiosis progression. Negative control siRNA or Bub3 siRNA was injected into GV stage oocytes and the injected oocytes were maintained in 2.5 µM milrinone for 12 hours (see details in Materials and Methods). We first detected the RNAi efficiency by Western blot. As presented in Fig. 5A, compared with the control groups (control siRNA injection or without injection), the Bub3 expression in siRNA-injected oocytes was significantly reduced, revealing successful Bub3 down-regulation by RNAi.

To further explore the role of Bub3 as a spindle checkpoint protein, we confirmed that the optimal dosage of nocodazole that was able to block the MI-AI transition but not GVBD in meiosis without apparent spindle damage was 0.04 µg/ml [32], [33] (Supplementary material Fig. S2 ruled out any side effects of nocodazole on meiosis I). Under this condition, 83.6% (97/116) of the oocytes were arrested at the MI stage. Then in the RNAi combined with nocodazole treatment experiment, oocytes with Bub3 RNAi were cultured in 0.04 µg/ml nocodazole for 12 hours. In the control group (with control siRNA injection), Bub3 signal was observed at kinetochores and 86.7% oocytes were arrested at the MI stage. Whereas in the Bub3 RNAi group, the Bub3 signal could not be detected and only 37.3% of the oocytes were arrested at the MI stage, while the others broke through the MI arrest, released PBI and reached the MII stage (Fig. 5B and C). These results revealed that down-regulation of Bub3 could override the metaphase arrest induced by nocodazole, indicating that Bub3 is essential for meiotic arrest of mouse oocytes in response to spindle damage.

### Deletion of Bub3 by RNAi causes misaligned chromosomes, abnormal polar bodies and aneuploidy

**Figure 6:**
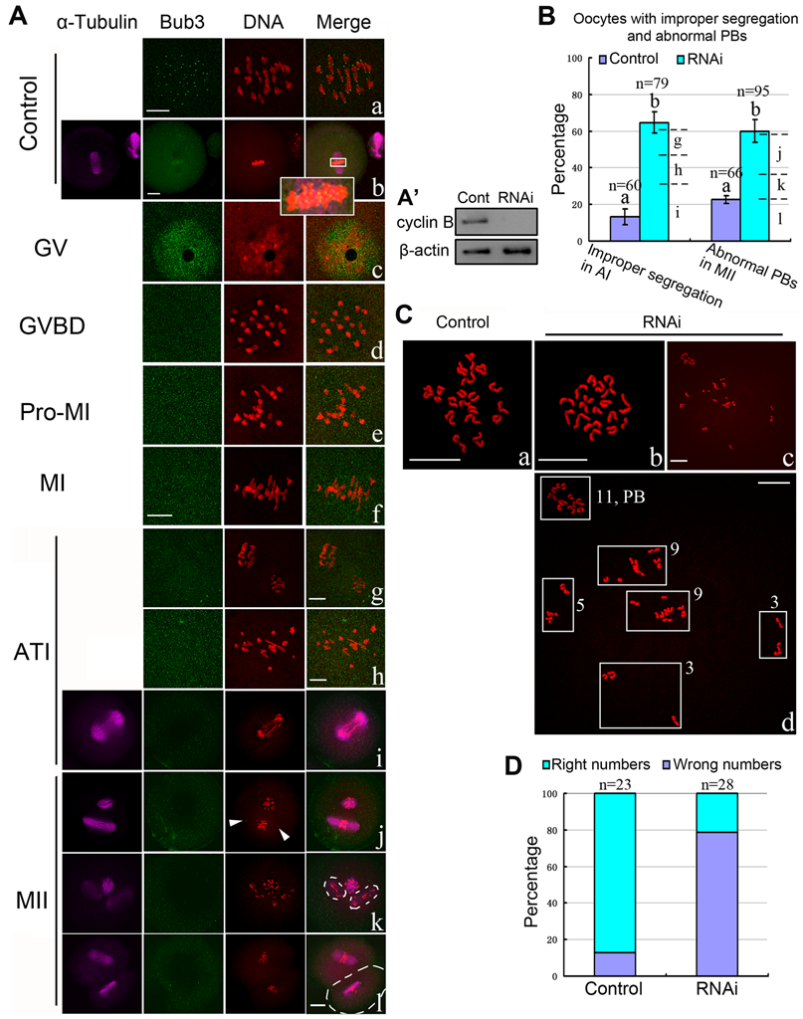
Bub3-RNAi leads to misaligned chromosomes, abnormal polar body, and aneuploidy. (A) Bub3-RNAi oocytes at various stages. Clear Bub3 signal was found at kinetochores in control groups (with control siRNA injection; (a), Pro-MI oocytes; (b), MII oocytes). No Bub3 signal was detected from GVBD to MII in RNAi groups. Anisomerous (g), straggled (h), or lagging chromosomes (i) were found during the AI stage. Misaligned chromosomes (j), double polar bodies (k), or enlarged polar body (l) were found in MII oocytes. Purple, α-tubulin; green, Bub3; red, DNA. Magnifications of the boxed regions are shown. Arrowhead indicates the misaligned chromosomes. Dot lines indicate the abnormal polar body. c–f share the same bar and i–l share the same bar. Bar  = 10 µm. (A′) Expression levels of cyclin B in MI stage oocytes (Cont) and AI stage oocytes (RNAi). Each sample contains 300 oocytes. (B) Percentage of oocytes with improperly separated chromosomes and abnormal PBs between the control and RNAi groups in AI and MII, respectively. Letters “g”–“l” correspond to the percentages of (g)–(l) in Fig. 6A. (C) and (D). Chromosome spreading was performed in control (with control siRNA injection) and RNAi oocytes. The numbers of univalents in the oocytes in Ca-Cc are 20, 18 and 29, respectively. “Cd” is the magnification of “Cc”. Bar  = 10 µm.

To further explore the detailed roles of Bub3, we analyzed the oocytes with Bub3 RNAi during meiosis. As shown in Fig. 6A, clear Bub3 signal was observed at kinetochores in the control group (with control siRNA injection) while no staining of Bub3 could be detected during Pro-MI and MII stages in RNAi groups. We subsequently calculated the rate of MI-AI transition and found that this event did not occur prematurely in the RNAi group compared with the control group (data not shown). However, at the AI stage, improper homologous chromosome segregation was observed in 65% of the oocytes, including anisomerous (Fig. 6Ag), straggled (Fig. 6Ah), or lagging chromosomes (Fig. 6Ai). To confirm that the oocytes with improper segregation in Fig. 6Ag-i did enter AI, another group of RNAi oocytes collected at the same time point and under the same culture conditions as those in Fig. 6Ag-i were subjected to Western blot to analyze the cyclin B level. As shown in Fig. 6A′, nearly no cyclin B was detected in RNAi oocytes, which was significantly different from that in control oocytes at the MI stage, suggesting that most oocytes derived from Fig. 6Ag-i entered AI. When the oocytes progressed to the MII stage, nearly 60% exhibited misaligned chromosomes (Fig. 6Aj), double polar bodies (Fig. 6Ak), or enlarged polar body (Fig. 6Al). The rates of improper segregation and abnormal polar bodies between the control (with control siRNA injection) and RNAi groups are presented in Fig. 6B. But the occurrence of PBE in the RNAi group did not advance compared with the control group (data not shown). To further prove the effects of RNAi, we used chromosome spreading experiments to determine the exact number of chromosomes in oocytes at the MII stage (Fig. 6C and D). As known for MII, the number of single chromosomes (univalents) in the mouse oocyte is 20 (control, Fig. 6Ca), which is the prerequisite for genomic integrity. Our results showed that RNAi oocytes typically displayed incorrect numbers (more or less than 20) of univalents (Fig. 6Cb and c). Considering the possible deviation derived from mechanical stress during chromosome spreading, and the possibility that even normal oocytes may have more or less than 20 chromosomes, we conducted chromosome spreading with exact chromosome numbers not only in oocyte but also in polar body. As shown in Figure 6Cc and d (“d” is an enlargement of “c”), the numbers of chromosomes in the oocyte and polar body were 29 and 11 (total of 40), respectively, verifying the credibility of RNAi-induced aneuploidy.

## Discussion

The major components of SAC include Mad1–2, BubR1 (Mad3 in yeast), Bub1, Bub3 and Mps1. It is generally accepted that the SAC proteins function as a complex and that they are interdependent [28], [34], [35], [36], [37], [38]. However, the individual role of Bub3, particularly in mammalian meiosis, has only been little explored. Furthermore, the existence of SAC in oocytes is still being debated [10], [21], [39], [40], [41]. Representative evidence against SAC function comes from studies using XO female mice, which harbor one univalent X chromosome that can not be properly attached but can be segregated at random in meiosis I without causing MI arrest [22], [42]. In our study, we found that endogenous Bub3 starts to localize at kinetochores in Pro-MI stage and maintains the localization until the MI-AI transition. Fig. 1Bd and e show an interesting behavior of Bub3 from “on ” to “off”, indicating that the retreat from kinetochores is a prerequisite for the oocytes to enter AI, which is consistent with the SAC proteins' feature in mitosis [3], [43] and meiosis [44]. Various studies proposed that some checkpoint proteins (Mad1, Mad2, Bub1, and Bub3) are present only at unattached rather than at fully microtubule-attached kinetochores [17], [18], [19], [20]. However, in mouse oocytes, we found that all the kinetochores display positive signal of Bub3 even though most of the homologues are well attached (Fig. 1Bd and 1Bb′). Moreover, early studies revealed that Bub1 in mouse oocytes and Bub3 in PtK_2_ cells were detectable at kinetochores during anaphase in meiosis and mitosis, respectively [45], [46], which is distinct from the Bub3 localization in our study (Figure 1Be, f). These differences may be due to the fact that although both Bub1 and Bub3 are key members of SAC, their individual behavior differs. Bub1 is required for the recruitment of other proteins to the kinetochores and to function as a scaffolding protein [47], [48] and it resides stably at the kinetochores which is significantly different from the rapidly cycled Bub3 [46]. Alternatively, one checkpoint protein does not function in the same manner in meiosis compared to mitosis. At the MII stage, Bub3 returns to kinetochores, which is similar to the localization of Mad2 and Bub1 [45], [49], implying that Bub3 has a potential function during the MII-AII transition (see details below).

To characterize the role of Bub3 in meiosis, we conducted overxpression experiments. Overexpressed Bub3 evidently inhibited the homologous chromosome segregation, indicating that Bub3 is involved in monitoring the MI-AI transition, which is similar to a previous report firstly showing *Xenopus* Bub3 requirement for spindle checkpoint activation and for maintenance of the spindle checkpoint signal [50]. In our study, this inhibition could last for at least 2 hours because oocytes in Fig. 2Bg, Fig. 2C, and Fig. 2C′ (Cont1 and OverE) were all cultured for 10 hours, two hours longer than that for oocyte entrance of the MI stage. In the overexpressed oocytes, the force keeping the homologues together is more powerful than the pulling force produced by spindle poles because in Fig. 2Bf-g, we can clearly observe the highly tense status of the homologues. By testing cyclin B levels, we proved that these overexpressed oocytes did stay at the MI stage (Fig. 2C′), implying the strictness of overexpressed Bub3 in monitoring the MI-AI transition. Different from the report in XO mice [22], [42], through chromosome spreading, we found that all chromosomes in the oocytes which were arrested in MI by overexpressed Bub3 were presented as bivalents without any univalents (Fig. 2D), implying that the SAC in meiosis may control chromosome segregation in an “all or none” manner. This is consistent with mitosis in which even a single unattached kinetochore can prevent the anaphase onset [51]. However, overexpression experiments may not completely reflect the function of endogenous Bub3 because a recent review pointed out that meiotic SAC triggering is not as stringently coupled to the kinetochore attachment of MTs as in mitosis and a lack of sensitivity or failure to respond to unattached kinetochores could account for the meiotic I error in females [10], indicating that different systems of spindle assembly pathways may exist between the meiosis and mitosis [52].

In a small number of oocytes which could not be inhibited at MI but entered AI, overexpressed signal was observed between the separated homologous chromosomes but not at the kinetochores (Fig. 2Eb). Indeed, not all the oocytes can express the exogenous Bub3-mRNA smoothly or in time (before the start of MI), so the translated protein can not always be recruited to the kinetochores at the right time which results in an accumulation at the mid-body. To some extent, this result supported an effect of overexpressed Bub3: the localization of overexpressed Bub3 at kinetochores and entry into AI did not occur in the same oocyte, which was a strict rule in our study. Among the oocytes which could overcome the inhibition and enter MII, a few exhibited an interesting chromosome status in that all the homologous chromosomes were pulled into the PB as shown in Fig. 2Ec, indicating that Bub3 play a more important role in monitoring chromosome segregation than PBE, which could also explain why Bub3-RNAi did not advance the occurrence of PBE.

Mammalian oocytes are arrested at the MII stage under control of the cytostatic factor (CSF) [53], [54]. Using *Xenopus* egg extracts, Bub1 was found to be required for CSF activity [55], which established a link between SAC and CSF. Further evidence showed that overexpressed Cdc20 could release *Xenopus* oocytes from meiosis II arrest [56]. However, using a Bub1 mutant, previous work by Tsurumi et al. demonstrated that CSF-arrested oocytes were not regulated by spindle checkpoint protein [57]. In the present study, we showed that Bub3 was involved in meiosis II because overexpressed Bub3 inhibited the MII-AII transition by preventing sister chromatid segregation (Fig. 3), indicating that Bub3 may be required for CSF in mouse oocytes. Indeed, the Bub1 mutant used in the study by Tsurumi et al. was reported to act as a dominant negative by competing with the kinetochore localization of the endogenous protein but did not deplete the endogenous protein and therefore it is possible that this Bub1 mutant did not interfere with a kinetochore-independent function required for CSF establishment or maintenance [57]. Moreover, the fact that Mad2 and Bub1 are localized at kinetochores in MII arrested oocytes [45], [49] supports a potential function of SAC proteins in CSF. It appears that different monitoring systems exist in meiosis I and II. Bub3 is a component of the MCC which functions as the inhibitory factor of APC/C to inhibit the MI-AI transition [1], [3]. While in meiosis II, Bub3 or SAC is involved in establishment of the CSF activity. In our RNAi experiment, however, depletion of Bub3 did not enable oocytes to progress into AII, indicating that down-regulation of single SAC protein is not sufficient to make an essential change to CSF activity. Indeed, various factors are required for CSF activity including Mos/MEK/MAPK/p90Rsk, Cdk2/cyclinE, RINGO, Emi1 [57], [58]. Cumulative evidence has revealed that the establishment of CSF on entry into meiosis II appears independent of its maintenance until fertilization [58]: immunodepletion of downstream components of the MAPK pathway, such as p90Rsk or xBub1, from CSF extracts does not cause the immediate release of these extracts from metaphase arrest, even though both are required to establish the CSF arrest [48], [55], [59]; addition of the potent inhibitor of Cdk2-cyclin E complexes to CSF extracts causes the complete inhibition of endogenous Cdk2-cyclin E activity, but is insufficient to release these extracts from metaphase arrest [55], indicating that these factors mediate the establishment of CSF but not its maintenance.

A previous study showed that DYNLT3 light chain directly links dynein to Bub3 [60], suggesting that dynein together with Bub3 is involved in the formation of stable bipolar kinetochore attachments to MTs. Recent work by other lab has also shown that like BubR1 and Bub1, Bub3 is required for proper chromosome-spindle attachment in mitosis [30]. During the MI-AI transition or AI stage, sister kinetochores face the same pole and are captured by MTs to form K-MTs so that homologous chromosomes move towards the opposite poles. Through overexpression of Bub3 during the MI-AI transition and cold treatment, we have provided evidence that Bub3 is involved in the kinetochore and microtubule interaction in meiosis (Fig. 4). Arrowheads in Fig. 4 Ab and Fig. 4 Ac indicate the misaligned chromosomes during the transition, which leads to the abnormal segregation. We believe that AI oocytes tend to be sensitive to the overexpressed Bub3 because endogenous Bub3 drops to the lowest level from this stage (Fig. 1A) and no signal can be detected at kinetochores (Fig. 1B) and Bub3 or SAC proteins are considered to be inactive at this time [3], [10], [43]; compulsively localized Bub3 at kinetochores by overexpression would disturb the attachment signal. It appears that shortly before the completion of Myc_6_-Bub3 mRNA expression, homologous chromosomes are aligned properly and ready to enter the AI stage. When Myc_6_-Bub3 mRNA translation is completed, the overexpressed Bub3 subsequently interferes with kinetochore-microtubule attachments so that these homologues move out of order during the segregation. A recent study has revealed a crucial complex in kinetochores called KMN which can bind microtubules and checkpoint proteins (especially binding Bub3 directly) in corresponding checkpoint “off” and “on”, respectively [61], indicating a competitive relationship between microtubules and Bub3.

By down-regulating Bub3 using RNAi, we showed that depletion of Bub3 abrogates the metaphase arrest induced by nocodazole, further indicating that Bub3 functions as a checkpoint protein, which is consistent with previous studies [30], [62], [63]. Additionally, we found that Bub3-RNAi caused misalignment of chromosomes, improper segregations, or abnormal polar bodies (Fig. 6A and B), which was similar to that in Mad2-depleted oocytes exhibiting large or multiple polar bodies [64]. However, the advance of the MI-AI transition and PBE did not occur in the RNAi group, indicating that Bub3 may not be as effective as the other members such as Mad2, Bub1, or BubR1 in SAC. Among these proteins, Mad2 and Mad3 (BubR1 in humans) have been shown to bind Cdc20 directly [65], [66]; BubR1 depletion leads to extensive misalignment defects that are more severe than those induced by Bub3 and Bub1 depletions [30]; loss of Bub1 in oocytes of Zp3-Cre transgenic mice greatly accelerates resolution of chiasmata and PBE, and the peak of APC/C activity in these oocytes is advanced by 5 hours [67]. On the other hand, SAC proteins have been reported to exert their function interdependently, and down-regulation of a single SAC protein therefore appears to be insufficient to advance the MI-AI transition and PBE. A dynamics study on checkpoint proteins revealed that Bub3 cycled through kinetochores much faster while Mad1 and Bub1 resided stably at kinetochores [46], indicating a Bub3 pool in the cytoplasm, which could buffer the down-regulation. On the other hand, due to the lager volume, the sensitivity of oocyte is not as the same as that of somatic cells. Using chromosome spreading, we found that most of the RNAi-oocytes exhibited incorrect numbers of chromosomes (Fig. 6C), which provides direct evidence for aneuploidy. An early study showed that in *Drosophila*, Bub3 was required for checkpoint-dependent mitotic arrest as its loss either by mutation or by RNAi causes precocious sister chromatid segregation, abnormal anaphase organization, and significant aneuploidy [68]. Deletion of Bub3 in the mouse is lethal early in embryogenesis with cells accumulating mitotic errors [62], [69]. Notably, meiosis in humans is particularly error-prone and the majority of the errors in oocytes originate during meiosis I [12], [70]. All the clues therefore reveal that Bub3 depletion is one critical cause leading to aneuploidy and up-regulation of some checkpoint proteins in oocytes with SAC deficiency may be a feasible strategy to overcome aneuploidy. In summary, although the characteristics of cell cycle in meiosis are different from those in mitosis, as a core member of SAC, Bub3 is required for monitoring the chromosome segregation in mammalian oocytes.

## Materials and Methods

All chemicals and media were purchased from Sigma Chemical Company (St. Louis, MO) except for those specifically mentioned.

### Oocyte collection and culture

ICR mice care and handling were conducted in accordance with policies promulgated by the Ethics Committee of the Institute of Zoology, Chinese Academy of Sciences. Oocytes were collected in M2 medium supplemented with 2.5 µM milrinone [71] to keep them at the germinal vesicle (GV) stage. After specific treatment, oocytes were washed thoroughly and cultured in M16 supplemented with 10% fetal bovine serum (FBS) (Gibco) to GV (0 hour), GVBD (2 hours), Pro-MI (5 hours), MI (8 hours), ATI (9.5 hours), and MII (12–14 hours) stages. The MII oocytes were released from CSF arrest by using 10 mM SrCl2 in Ca^2+^/Mg^2+^ -free CZB.

### Nocodazole and cold treatment of oocytes

For nocodazole treatment, 10 mg/ml nocodazole in DMSO stock was diluted in M16 medium to give a final concentration of 0.04 µg/ml. Oocytes at the appropriate stages were incubated in the M16 medium containing 0.04 µg/ml nocodazole for 12 hours. After treatment, oocytes were washed thoroughly and used for immunofluorescent staining; for the recovery experiment (see Fig. S2 in supplementary material), nocodazole-treated oocytes were subsequently washed thoroughly followed by culture in fresh culture medium for 2 hours. Control oocytes were treated with the same concentration of DMSO in the medium before examination. For cold treatment, oocytes were first cultured for 7 hours and then injected with Myc6-Bub3 or control Myc6 mRNA followed by additional 2 hours culture when most of the oocytes expressed the protein and were undergoing or just completed the MI-AI transition. Then the oocytes were transferred to M16 medium which was precooled at 4°C, and were cultured for 25 minutes at this temperature, followed by immunofluorescent staining.

### Bub3 plasmid construction

Total RNA was extracted from 150 mouse GV oocytes using RNeasy micro purification kit (Qiagen), and the first strand cDNA was generated with cDNA synthesis kit (Takara), using poly (dT) primers. The following two nested primers were used to clone the full length of Bub3 cDNA by PCR. F1: GGAAGCGGATCGGTAGTGG, R1: CTGGCTGAAAGAAGTCAAGTGG, F2: TCAGGCCGGCCGATGACCGGTTCGAACGAATTC, R2: GTTGGCGCGCCTCACGTGGACTTGGGCTTTGTT. To detect the overexpressed protein, the Bub3 cDNA was then NH_2_-terminally Myc_6_-tagged. For *in vitro* transcription reactions, the Myc_6_-Bub3 cDNA was subcloned into the modified pRN3p vector (a gift from Dr. Jie Na, Harvard University), which has a globin 3′ UTR plus a short poly A tail.

### RNA synthesis

The Myc_6_-Bub3-pRN3p plasmid was linearized by S*fi*I and purified by gel extraction kit (Qiagen). T3 message machine (Ambion) was used to produce capped mRNA which was purified using the RNeasy cleanup kit (Qiagen). The concentration was detected by Beckman DU 530 Analyzer, and diluted into 2.5 mg/ml for overexpression of the protein.

### Microinjection of Myc_6_-Bub3 mRNA or Bub3 siRNAs

Microinjections were performed using a Nikon Diaphot ECLIPSE TE 300 (Nikon UK Ltd., Kingston upon Thames, Surrey, UK) inverted microscope equipped with Narishige MM0-202N hydraulic three-dimensional micromanipulators (Narishige Inc., Sea Cliff, NY) and completed within 30 minutes. For Myc_6_-Bub3 overexpression, 2.5 mg/ml Myc_6_-Bub3 mRNA solution was injected into cytoplasm of the oocytes and expression of Myc_6_-Bub3 protein was completed within 2–2.5 hours. The same amount of H_2_O or Myc_6_ mRNA (virtually no discrepancy was observed between them) was injected as control. Each experiment consisted of three separate replicates and approximately 100 oocytes were injected in each group. The ratio of GVBD or PBE was determined by counting using an inverted optical microscope at the appropriate time points. For MII oocyte overexpression experiments, 2.5 mg/ml Myc_6_-Bub3 mRNA was injected into the cytoplasm and the oocytes were cultured for 2 hours before they were parthenogenetically activated by 10 mM SrCl_2_.

Small interfering RNAs (siRNAs) of Bub3 siRNAs (Ambion) were microinjected into the cytoplasm to deplete Bub3. 25 µM Bub3 siRNA was used: UGCACGAUUUGAACACUGAtt. The same amount of negative control siRNA (Qiagen) was injected as control. After microinjection, the GV oocytes were cultured for 12 hours in M16 supplemented with 2.5 mM milrinone to prevent meiosis resumption. We confirmed the prolonged GV-arrested oocyte quality by morphological evaluation under the optical microscope and chromosome spreading. The rates of GVBD and extrusion of the first polar body (PBE) were not statistically different from the normal cultured oocytes without inhibition (data not shown).

### Immunoblotting analysis

Mouse oocytes at the appropriate stages of meiotic maturation and oocytes injected with Myc_6_-Bub3 mRNA, Bub3 siRNA, control mRNA or control RNAi were collected in SDS sample buffer and boiled for 5 minutes. Immunoblotting was performed as described previously [72]. Briefly, the proteins were separated by SDS-PAGE and then electrically transferred to polyvinylidene fluoride membranes. Following transfer, the membranes were blocked in TBST (TBS containing 0.1% Tween 20) containing 5% skimmed milk for 2 hours, followed by incubation overnight at 4°C with anti-Bub3 (BD Biosciences), anti-myc (Invitrogen) or anti-cyclin B1 (BD Biosciences) with dilutions of 1∶500, 1∶1000, and 1∶500, respectively. After washing in TBST, the membranes were incubated for 1 hour at 37°C with 1∶1000 horseradish peroxidase (HRP) -conjugated IgG. To detect β-actin, the membranes were washed in the washing buffer (100 mM β-mercaptoethanol, 20% SDS, and 62.5 mM Tris, pH 6.7) for 30 minutes at 55°C. β-actin was then assayed on the same membrane by using anti-β-actin antibody (1∶1000) and HRP-conjugated IgG. Finally, the membranes were detected by the enhanced chemiluminescence detection system (Amersham, Piscataway, NJ).

### Immunofluorescent microscopy, chromosome spreading and image analysis

Immunofluorescence was performed as described previously [33]. For single staining of Bub3, Myc_6_-Bub3, and α-tubulin, oocytes were fixed in 4% paraformaldehyde in PBS (pH 7.4) for at least 30 minutes at room temperature. After being permeabilized with 0.5% Triton X-100 at room temperature for 20 minutes, oocytes were blocked in 1% BSA-supplemented PBS for 1 hour and incubated overnight at 4°C with 1∶50 anti-Bub3 (BD Biosciences), 1∶200 anti-Myc-FITC (Invitrogen), or 1∶200 anti-α-tubulin-FITC antibodies, respectively. After three washes in PBS containing 0.1% Tween 20 and 0.01% Triton X-100 for 5 minutes each, the oocytes were labeled with 1∶100 FITC-conjugated IgG for 1 hour at room temperature (for staining of Myc and α-tubulin, this step was omitted). After washing in PBS containing 0.1% Tween 20 and 0.01% Triton X-100, the oocytes were co-stained with propidium iodide (PI; 10 µg/ml in PBS) or Hoechst 33258 (10 µg/ml in PBS). Finally, the oocytes were mounted on glass slides and examined with a confocal laser scanning microscope (Zeiss LSM 510 META, Germany). For chromosome spreading, oocytes were left for 15 minutes in 1% sodium citrate at room temperature and then fixed with fresh methanol: glacial acetic acid (3∶1). 10 mg/ml PI was used for chromosome staining. Cells were examined with a Confocal Laser Scanning Microscope (Zeiss LSM 510 META, Germany). Instrument settings were kept constant for each replicate.

### Statistics

All percentages from at least three repeated experiments were expressed as means ± SEM and analyzed by ANOVA using SPSS software (SPSS Inc, Chicago, IL) followed by the student-Newman-Keuls test. *P*<0.05 was considered statistically significant.

## Supporting Information

Figure S1 Scanned oocytes in consecutive planes. Oocyte with PBI was scanned in numerous planes to determine whether there were chromosomes in the oocyte. In all the planes, chromosomes were detected only in the PBI but not the oocyte. Red, overexpressed Bub3; blue, DNA (chromosomes). Bar  = 10 µm.
(0.76 MB TIF)

Figure S2 Oocytes' recovery from the treatment of low dose of nocodazole in meiosis I. Control group, GV oocytes were cultured for 5 hours without nocodazole when oocytes progressed to the Pro-MI stage and normal spindles were observed. Nocodazole group, GV oocytes were cultured for 5 hours with 0.04 µg/ml nocodazole. Feeble but still existing spindle was observed. Recovery group, GV oocytes were cultured for 5 hours with 0.04 µg/ml nocodazole followed by thorough washes and then cultured in fresh culture medium for 2 hours. Oocytes were allowed to recover in the fresh culture medium and progressed to the stage close to MI with normal spindle. Bar  = 10 µm.
(2.55 MB TIF)
