# Supplementary figures and images for: Bub3 Is a Spindle Assembly Checkpoint Protein Regulating Chromosome Segregation during Mouse Oocyte Meiosis
Source: PLoS One. 2009 Nov 2;4(11):e7701. doi: 10.1371/journal.pone.0007701 (PMC2765619; doi:10.1371/journal.pone.0007701)

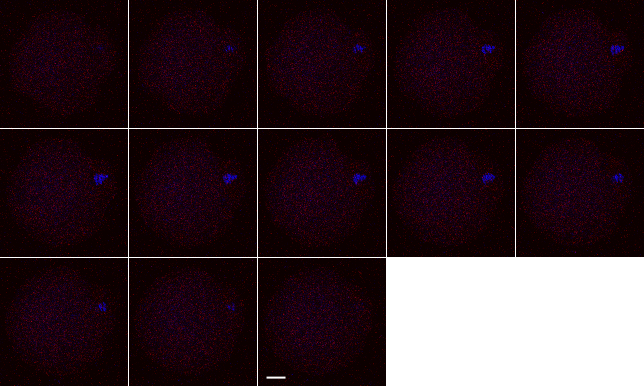

Supplement: Figure S1 — Scanned oocytes in consecutive planes. Oocyte with PBI was scanned in numerous planes to determine whether there were chromosomes in the oocyte. In all the planes, chromosomes were detected only in the PBI but not the oocyte. Red, overexpressed Bub3; blue, DNA (chromosomes). Bar = 10 µm. (0.76 MB TIF) [file pone.0007701.s001.tif]

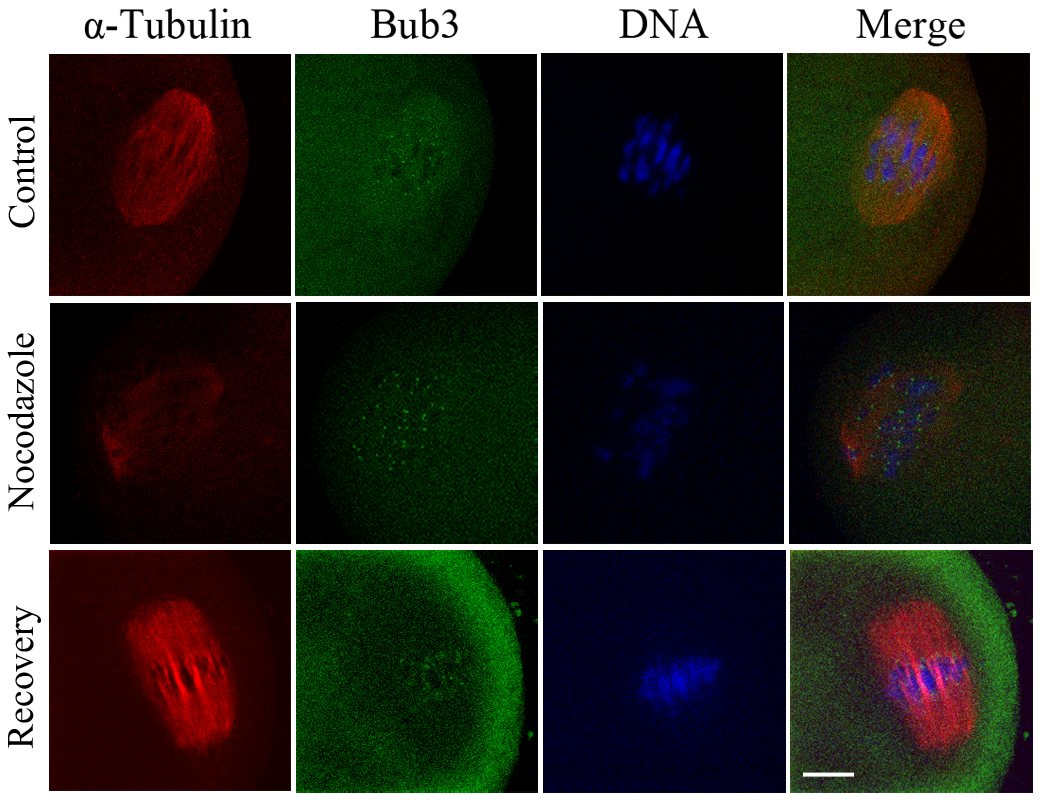

Supplement: Figure S2 — Oocytes' recovery from the treatment of low dose of nocodazole in meiosis I. Control group, GV oocytes were cultured for 5 hours without nocodazole when oocytes progressed to the Pro-MI stage and normal spindles were observed. Nocodazole group, GV oocytes were cultured for 5 hours with 0.04 µg/ml nocodazole. Feeble but still existing spindle was observed. Recovery group, GV oocytes were cultured for 5 hours with 0.04 µg/ml nocodazole followed by thorough washes and then cultured in fresh culture medium for 2 hours. Oocytes were allowed to recover in the fresh culture medium and progressed to the stage close to MI with normal spindle. Bar = 10 µm. (2.55 MB TIF) [file pone.0007701.s002.tif]
